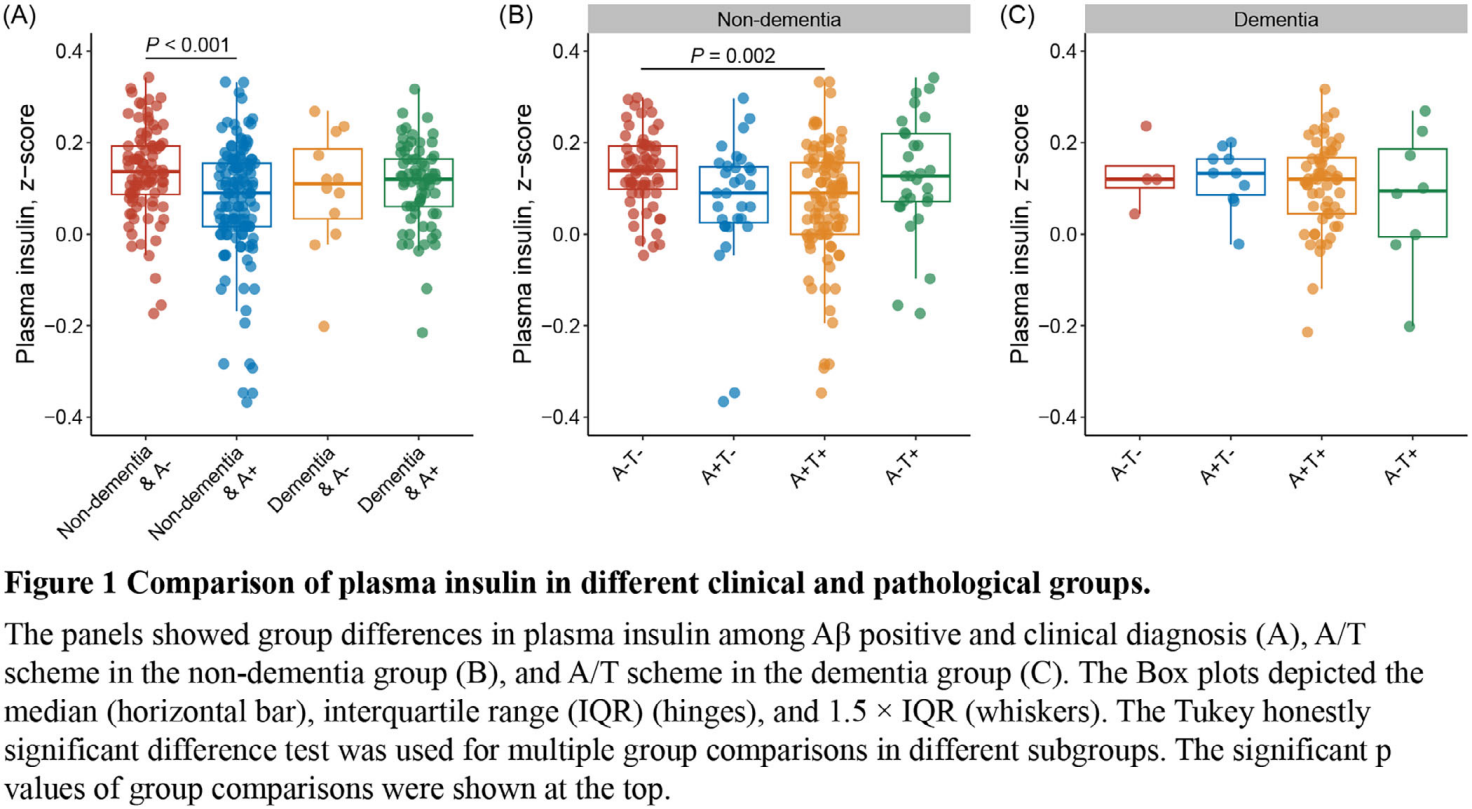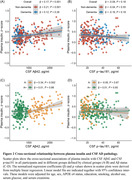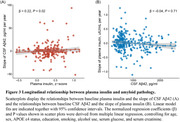# Plasma insulin is an early biomarker of amyloid‐β pathology in Alzheimer’s disease

**DOI:** 10.1002/alz.084870

**Published:** 2025-01-09

**Authors:** Yuhan Chen, Zhibo Wang, Zhiqi Mao

**Affiliations:** ^1^ Hebei North University, Zhangjiakou, Hebei China; ^2^ Xuanwu Hospital, Capital Medical University, Beijing, Beijing China; ^3^ Chinese PLA General Hospital, Beijing, Beijing China

## Abstract

**Background:**

Evidence suggests that type 2 diabetes (T2D) is an independent risk factor for Alzheimer's disease (AD), sharing similar pathophysiological traits like impaired insulin signaling. However, how plasma insulin changes with AD key pathologies and its diagnostics value remain unclear.

**Method:**

A total of 304 participants were included in the Alzheimer’s Disease Neuroimaging Initiative (ADNI), assessing plasma insulin and cerebrospinal fluid (CSF) AD pathology. We explored the cross‐sectional and longitudinal associations between plasma insulin and AD pathology and compared their associations across different AD clinical and pathological stages. We also explored the potential diagnostic value of plasma insulin on AD pathology using the receiver operating characteristic (ROC) curves.

**Result:**

In the non‐demented group, A+ participants (e.g., as reflected by CSF Aβ42) exhibited significantly reduced plasma insulin levels compared to non‐demented A‐ participants (P < 0.001). Furthermore, A+T‐ participants (e.g., as reflected by CSF Aβ42 and CSF p‐tau181) had lower plasma insulin levels than A‐T‐ participants in the non‐dementia group (P = 0.002). No significant differences were observed in the dementia group (Figure 1). In addition, a notable positive correlation was found between plasma insulin levels and CSF Aβ42 (P < 0.001) in all participants. When stratified by clinical status and pathological status, this association only existed in the A‐ group (P = 0.002) and the non‐demented group (P < 0.001) (Figure 2). Moreover, plasma insulin at baseline was significantly associated with longitudinal changes in CSF Aβ42 (P = 0.02), while baseline CSF Aβ42 was not associated with longitudinal changes in plasma insulin (Figure 3). Regarding the diagnostical value, plasma insulin alone demonstrated acceptable accuracy in distinguishing A+ from A‐ individuals in the whole participants (area under the curve [AUC]=0.61) and more prominent in the non‐dementia group (AUC = 0.65) than in the dementia group (AUC = 0.52). When added age, sex, and APOE ε4 status were as predictors, the AUC of plasma insulin reached 0.8 in the whole participants.

**Conclusion:**

These findings pinpoint the association between plasma insulin and early Aβ pathology in the early stages of AD, suggesting that plasma insulin can serve as an early biomarker of Aβ pathology.